# Diabetic retinopathy and the risk of all-cause dementia, Alzheimer’s disease, and vascular dementia: a systematic review and meta-analysis

**DOI:** 10.3389/fmed.2026.1716648

**Published:** 2026-06-30

**Authors:** Haoshen Hu, Yue Wang, Yidan Xu, Lixun Chen

**Affiliations:** 1 Department of Ophthalmology, Nanjing First Hospital, Nanjing Medical University, Nanjing, China; 2 Nanjing Red Cross Eye Bank, Nanjing, China

**Keywords:** Alzheimer’s disease, dementia, diabetic retinopathy, meta-analysis, systematic review, vascular dementia

## Abstract

**Background:**

Existing evidence on the association between diabetic retinopathy (DR) and the risk of all-cause dementia (ACD), Alzheimer’s disease (AD), and vascular dementia (VD) remains inconsistent. To address this gap, we performed a systematic review and meta-analysis to quantitatively assess the relationship between DR patients and subsequent dementia risk, aiming to inform evidence-based prevention strategies.

**Methods:**

We systematically searched PubMed, Embase, and Web of Science for cohort studies investigating the association between DR and the risk of ACD, AD, and VD. The searches covered all available literature from the inception of database to September 2025. After literature screening, data extraction, and risk-of-bias assessment, meta-analysis was conducted using Stata 14.0. Pooled hazard ratios (HRs) and 95% confidence intervals (CIs) were calculated.

**Results:**

A total of 10 studies involving 1,720,128 participants were included. The meta-analysis showed that DR was significantly associated with higher risks of ACD (HR = 1.24, 95%CI [1.14, 1.36], *p* < 0.00001), VD (HR = 1.20, 95%CI [1.05, 1.37], *p* = 0.009), and AD (HR = 1.23, 95%CI [1.11, 1.37], *p* < 0.00001).

**Conclusion:**

Current evidence indicates that DR is associated with an elevated risk of ACD, AD, and VD. Clinically, early screening and regular monitoring of cognitive function should be strengthened in patients with DR, and these assessments should be integrated into comprehensive diabetes care. Given the inherent limitations of this study, further high-quality prospective cohort studies are needed to confirm these findings.

## Introduction

Diabetes mellitus (DM) has become a major global public health issue (1, 2). Chronic hyperglycemia causes a spectrum of long-term complications, encompassing macrovascular diseases and microvascular disorders such as diabetic nephropathy, diabetic retinopathy (DR), and diabetic neuropathy (3, 4). DR is one of the most prevalent microvascular complications in individuals with diabetes (5). Its pathogenesis is primarily driven by retinal vascular injury resulting from persistent hyperglycemia (6). As diabetes prevalence continues to increase, the incidence of DR increases annually, imposing a substantial burden on patients’ quality of life and potentially leading to severe sequelae, including blindness. In 2020, an estimated 103 million people worldwide were living with DR, and this figure is projected to reach 161 million by 2045 (7). Notably, emerging evidence indicates that DR may exert systemic effects beyond ocular involvement, contributing to the onset and progression of other systemic diseases (8–10).

Dementia is a neurodegenerative syndrome characterized primarily by cognitive decline (11). It includes multiple subtypes, such as all-cause dementia (ACD), Alzheimer’s disease (AD), and vascular dementia (VD) (12, 13). These disorders severely impair patients’ quality of life and functional independence, placing a heavy burden on families and society. As the global population ages, the incidence of dementia is rising steadily, and its pathogenesis has become a major research focus. Accumulating studies have examined the link between diabetes and dementia (14–16). Evidence indicates that individuals with diabetes face an elevated risk of dementia (17–19). Furthermore, as a key microvascular complication of diabetes, DR may also be associated with dementia development (20, 21). Investigating the relationship between DR and dementia is of theoretical importance for understanding the pathogenesis of diabetes-related cognitive impairment. Clarifying this relationship may help identify risk factors and pathological mechanisms driving cognitive decline in diabetic patients, providing a theoretical basis for developing novel therapeutic targets and intervention strategies.

Although most large-scale cohort studies have consistently confirmed a positive correlation between DR and dementia risk, the magnitude of this correlation varies considerably across studies, accompanied by substantial heterogeneity in effect sizes. Several studies (22–24) reported that DR patients face a markedly elevated dementia risk, while other investigations (21, 25) failed to detect a significant association between the two conditions. This study, through a systematic review and meta-analysis, aims to comprehensively evaluate the association between DR and the risks of ACD, AD, and VD, and to provide a quantitative synthesis of the current observational evidence on the magnitude and consistency of these associations.

## Methods

Inclusion criteria include.

(1) Study design: cohort studies;
(2) Research theme: studies investigating the associations between DR and the risks of ACD, AD, or VD had to be examined;
(3) Exposure definition: DR was clearly defined as study exposure;
(4) Outcome definition: ACD, AD, or VD was clearly identified as the study outcome;
(5) Data availability: studies that reported sufficient quantitative risk data, including hazard ratios (HRs) and corresponding 95% confidence intervals (CIs) for pooled analysis.

Exclusion criteria include.

(1) Duplicate publications and overlapping study data;
(2) Non-original studies, including systematic reviews, meta-analyses, editorial comments, conference abstracts, and case reports;
(3) Studies with unavailable or incomplete data that could not be extracted or converted for quantitative meta-analytic synthesis.

### Search strategy

We systematically searched PubMed, Embase, and Web of Science for cohort studies investigating the associations between DR patients and the risks of ACD, AD, or VD. The search period spanned from database inception to September 2025. Search strategies combined Medical Subject Headings (MeSH) terms and free words. The core search terms included “diabetic retinopathy,” “dementia,” “all-cause dementia,” “Alzheimer’s disease,” and “vascular dementia,” along with their relevant variants. Detailed search strategies for each database are presented in Supplementary Table S1.

### Study selection and data extraction

First, the retrieved literature was imported into EndNote. Duplicate articles were eliminated according to author names, publication years, and journal titles. Titles and abstracts were then screened to exclude studies that did not meet the preset inclusion and exclusion criteria. Full-text assessments were finally conducted to confirm the eligibility of studies.

Extracted data covered three aspects: (1) basic study information, including first author, publication year, study design, region, and data source; (2) population characteristics such as age, gender, and sample size; (3) outcome indicators referring to the association effect sizes between DR and risks of ACD, AD, and VD, namely HRs and corresponding 95% CIs.

Literature screening and data extraction were independently completed by two researchers. Discrepancies were settled via consultation with a third investigator.

### Assessment of risk of bias

Two researchers independently evaluated the risk of bias in the included studies using the Newcastle–Ottawa Scale (NOS). Discrepancies were resolved by consulting a third researcher. The NOS assesses studies in three dimensions: selection, comparability, and outcome assessment. Studies with scores of 7–9 were defined as high quality with low bias risk; scores of 4–6 indicated moderate quality and moderate bias risk; and scores of 0–3 represented low quality with high bias risk.

### Data analysis

All meta-analyses were performed using Stata 14.0 to evaluate the associations between DR patients and the risks of ACD, AD, or VD. The HR and its 95% CI were calculated. Between-study heterogeneity was assessed using the *χ*^2^ test and *I*^2^. A fixed-effects model was adopted when *p* > 0.10 and *I*^2^ < 50%, whereas a random-effects model was applied when *p* ≤ 0.10 and *I*^2^ ≥ 50% (26, 27). Subgroup analyses were further stratified by study design (retrospective vs. prospective), sample size (<10,000 vs. ≥10,000), geographical region (Asian vs. non-Asian), and adjustment for the APOE ε4 (adjusted vs. unadjusted). Sensitivity analysis was performed using the elimination method to determine the stability of the results. Egger’s test was utilized to detect potential publication bias.

## Results

### Study selection

**Figure 1:**
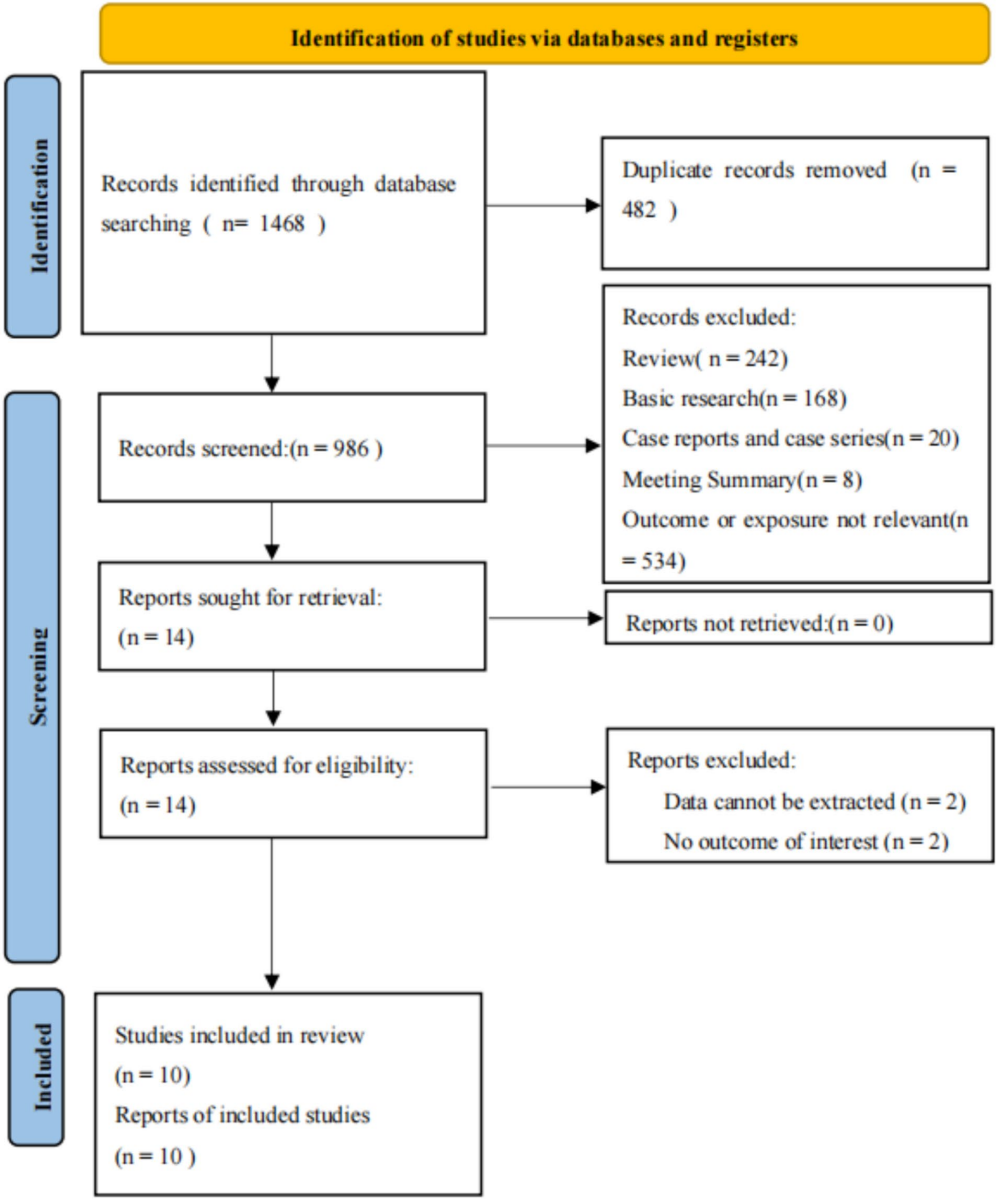
Flow chart of study selection and exclusion.

A total of 1,468 potentially relevant records were initially retrieved. After removing 482 duplicate records, 986 records remained. Following title/abstract screening, 972 records were excluded due to review (*n* = 242), basic research (*n* = 168), case reports and case series (*n* = 20), meeting summary (*n* = 8), and outcome or exposure not relevant (*n* = 534). The remaining 14 full-text articles were assessed for eligibility; 4 were excluded: 2 because data could not be extracted, and 2 because they lacked outcomes of interest. Finally, 10 eligible studies were included in the meta-analysis (20–25, 28–31) (Figure 1; Supplementary Table S2).

### Characteristics of included studies

The final analysis incorporated 10 studies, comprising a total of 1,720,128 participants. These studies were conducted in the United States, South Korea, Denmark, Netherlands, and China, and included three prospective and seven retrospective cohort studies. The sample size of individual studies ranged from 772 to 786,263 participants. All studies reported outcomes for ACD, AD, and VD. The NOS scores of the included studies ranged from 7 to 8, indicating a low risk of bias across all enrolled studies. Detailed baseline characteristics of the included studies are summarized in Table 1.

**Table 1:** Characteristics of the included studies.

Study	Country	Data source	Study design	No. of participants	Age	Women, %	Outcome	NOS score	Adjustments
Exalto et al. (22)	United States	Kaiser Permanente Northern California Diabetes Registry	Retrospective cohort study	29,961	70.6	13,787 (46.0%)	ACD	7	Age, sex, race, education, medical utilization, and diabetic medication use
Hwang et al. (25)	United States	Cardiovascular Health Study	Prospective cohort study	772	74.8	446 (57.77%)	ACD, AD, VD	8	Age, sex, race, education, cardiovascular, dementia, body mass index, smoking status, alcohol intake, physical activity level, total cholesterol level, diabetes mellitus status, hypertension status, history of cardiovascular disease, history of cerebrovascular disease, and apolipoprotein E ε4 allele, cohort, and clinic site
Lee et al. (28)	United States	Kaiser Permanente Washington	Retrospective cohort study	2,532	NR	1,412 (55.77%)	AD	7	Age, sex, education, self-reported white race, any APOE ε4 alleles, and time-dependent smoking status
Lee et al. (23)	Korea	Electronic health records	Retrospective cohort study	27,719	NR	NR	ACD, AD, VD	7	NR
Pedersen et al. (20)	Denmark	The Danish Health Registries	Prospective cohort study	786,263	≥60	442,666 (56.3%)	AD	7	Sex, age, depression, marital status, use of antihypertensive drugs, lipid-lowering drugs, and Charlson comorbidity index: myocardial infarction, congestive heart failure, cerebrovascular disease, chronic pulmonary disease, connective tissue disease and rheumatologic disease, ulcer disease, mild or moderate–severe liver disease, renal disease, hemiplegia or paraplegia, any malignancies, and acquired immunodeficiency syndrome
Rodill et al. (21)	United States	Kaiser Permanente Northern California database	Retrospective cohort study	3,742	56.1	1772 (47.4%)	AD	7	Baseline glycosylated hemoglobin (Baseline comorbidities include neuropathy, diabetic nephropathy, end-stage renal disease, cardiovascular disease, stroke, and hyperglycemic or hypoglycemic episodes) and comorbidities
Schrijvers et al. (29)	Netherlands	The Rotterdam Study	Prospective cohort study	6,273	72	3,721 (59.32%)	ACD, AD, VD	7	Age, sex, stroke, systolic blood pressure, use of antihypertensive medication, education, APOE ϵ4 carriership, current cigarette smoking, diabetes, total cholesterol, C-reactive protein, and coronary heart disease
Shang et al. (24)	China	UK Biobank cohort	Retrospective cohort study	90,524	62	48,141 (53.18%)	ACD	7	Age, sex, education, income, smoking, alcohol consumption, physical activity, Body Mass Index, cholesterol, glucose, and intake of cooked vegetables, raw vegetables, fresh fruits, and dried fruits at baseline
Yen et al. (30)	China	Health Insurance Research Database of Taiwan	Retrospective cohort study	576,893	NR	NR	ACD, AD, VD	7	Sex, age, obesity, smoking, alcohol-related disorders, co-morbidities, antidiabetic drugs, and cardiovascular-related drugs
Yu et al. (31)	Korea	National Health Insurance Service data	Retrospective cohort study	195,449	>40	NR	ACD, AD, VD	7	Age, sex, smoking, alcohol intake, exercise, income, plasma glucose concentration, duration of diabetes, body mass index, dyslipidemia, hypertension, diabetic retinopathy, chronic kidney disease, stroke, Ischemic heart disease, depression, number of oral hypoglycemic agents, and treatment with insulin

ACD, all-cause dementia; AD, Alzheimer’s disease; VD, vascular dementia; NOS, Newcastle–Ottawa Scale; NR, no report.

### Risk of all-cause dementia

**Figure 2:**
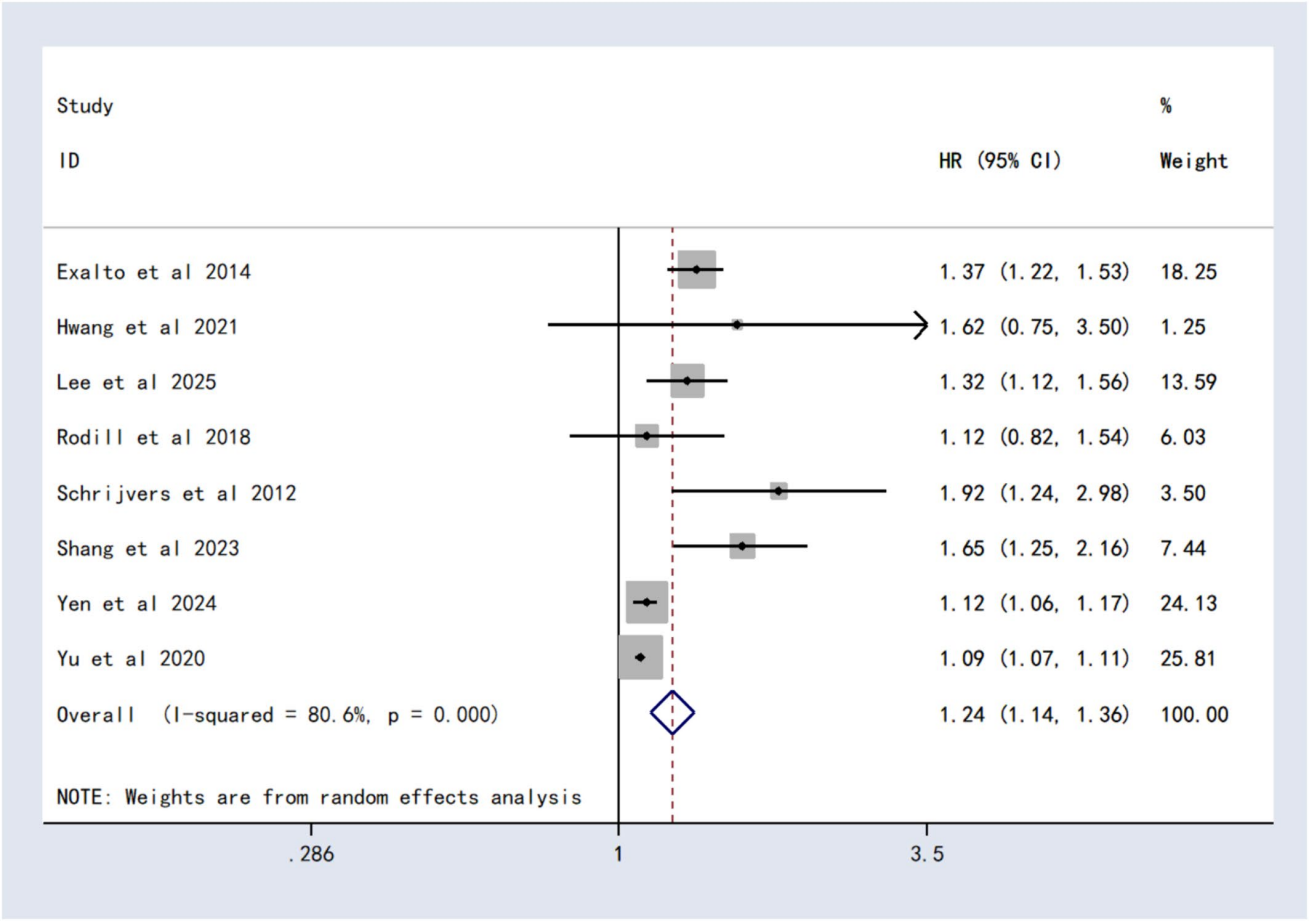
Meta-analysis of the hazard ratios of the association between diabetic retinopathy and all-cause dementia.

Eight studies explored an association between DR patients and the risk of ACD. Significant heterogeneity was observed across the included studies (*I*^2^ = 80.6%, *p* < 0.00001); thus, a random-effects model was applied. The pooled results revealed that DR was significantly associated with an increased risk of ACD (HR = 1.24, 95%CI [1.14, 1.36], *p* < 0.00001) (Figure 2; Table 2).

**Table 2:** Pooled results of each outcome.

Outcomes	No. of study	Heterogeneity		Meta-analysis		Publication bias	
		*I* ^2^	*p*	HR (95%CI)	*p*	Begg’s test (*p*)	Egger’s test (*p*)
All-cause dementia	8	80.60%	<0.00001	1.24 (1.14, 1.36)	<0.00001	0.707	0.058
Vascular dementia	5	63.90%	0.026	1.20I (1.05, 1.37)	0.009	0.308	0.544
Alzheimer’s disease	7	69.00%	0.004	1.23 (1.11, 1.37)	<0.00001	0.707	0.087

### Risk of vascular dementia

**Figure 3:**
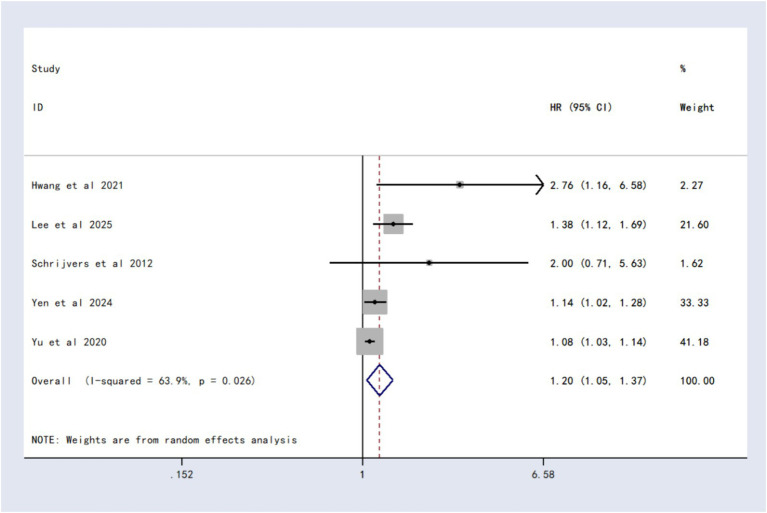
Meta-analysis of the hazard ratios of the association between diabetic retinopathy and vascular dementia.

Five studies reported the association between DR patients and the risk of VD. Moderate-to-high heterogeneity was detected (*I*^2^ = 63.9%, *p* = 0.026); therefore, a random-effects model was used. The meta-analysis demonstrated that DR significantly elevated the risk of VD (HR = 1.20, 95%CI [1.05, 1.37], *p* = 0.009; Figure 3; Table 2).

### Risk of Alzheimer’s disease

**Figure 4:**
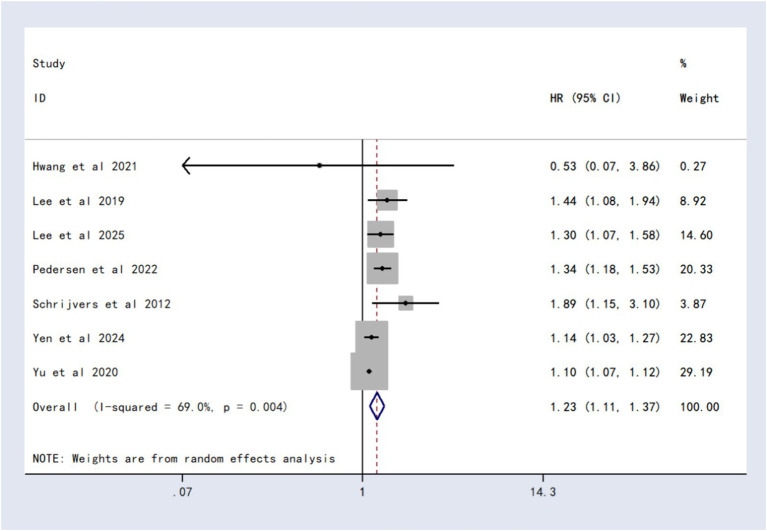
Meta-analysis of the hazard ratios of the association between diabetic retinopathy and Alzheimer’s disease.

Seven studies assessed the association between DR patients and the risk of AD. Substantial between-study heterogeneity was identified (*I*^2^ = 69.0%, *p* = 0.004), warranting the use of a random-effects model. The pooled analysis indicated that DR significantly increased the risk of developing AD (HR = 1.23, 95%CI [1.11, 1.37], *p* < 0.00001) (Figure 4; Table 2).

### Subgroup analysis

Subgroup analyses were performed according to study design (retrospective vs. prospective), sample size (<10,000 vs. ≥10,000), geographic region (Asian vs. non-Asian), and adjustment for the APOE ε4 mutation (adjusted vs. unadjusted). The results consistently demonstrated that DR was significantly associated with an increased risk of ACD, AD, and VD across all stratified subgroups (Table 3). The study design (prospective and retrospective), sample size, geographical region, and whether APOE ε4 was adjusted were all potential sources of heterogeneity. However, these heterogeneities did not affect the stability of the meta-analysis results.

**Table 3:** Subgroup analysis of each outcome.

Subgroup analysis	No. of studies	Heterogeneity		HR (95%CI)	*p*
		*I* ^**2**^	*p*		
All-cause dementia					
Study design					
Retrospective	6	82.70%	<0.00001	1.21 (1.12,1.32)	<0.00001
Prospective	2	0.00%	0.707	1.84 (1.26,2.70)	0.002
Sample size					
<10,000	3	50.40%	0.133	1.37 (1.08,1.75)	0.011
>10,000	4	86.20%	<0.00001	1.22 (1.12,1.34)	<0.00001
Region					
Asian	3	66.20%	0.052	1.12 (1.06,1.18)	<0.00001
Non-Asian	5	28.40%	0.232	1.10 (1.08,1.12)	<0.00001
APOE ε4					
Yes	2	0.00%	0.707	1.84 (1.26,2.70)	0.002
No	6	82.70%	<0.00001	1.21 (1.12, 1.32)	<0.00001
Vascular dementia					
Study design					
Retrospective	3	0.64%	0.063	1.10 (1.05,1.15)	<0.00001
Prospective	2	0.00%	0.64	2.42 (1.24,4.70)	0.009
Sample size					
<10,000	2	0.00%	0.64	2.42 (1.24,4.70)	0.009
>10,000	3	63.90%	0.063	1.15 (1.03,1.28)	0.011
Region					
Asian	3	63.90%	0.063	1.15 (1.03,1.28)	0.011
Non-Asian	2	0.00%	0.64	2.42 (1.24,4.70)	0.009
APOE ε4					
Yes	2	0.00%	0.64	2.42 (1.24,4.70)	0.009
No	3	63.90%	0.063	1.15 (1.03,1.28)	0.011
Alzheimer’s disease					
Study design					
Retrospective	4	52.40%	0.098	1.11 (1.08,1.13)	<0.00001
Prospective	3	22.70%	0.274	1.42 (1.09,1.85)	0.009
Sample size					
<10,000	3	0.00%	0.38	1.52 (1.18,1.95)	0.001
>10,000	4	73.80%	0.01	1.19 (1.08,1.31)	<0.00001
Region					
Asian	3	36.70%	0.206	1.10 (1.08,1.13)	<0.00001
Non-Asian	4	0.00%	0.441	1.37 (1.23,1.54)	<0.00001
APOE ε4					
Yes	3	0.00%	0.38	1.52 (1.18,1.95)	0.001
No	4	73.80%	0.01	1.19 (1.08,1.31)	<0.00001

### Sensitivity analysis

**Figure 5:**
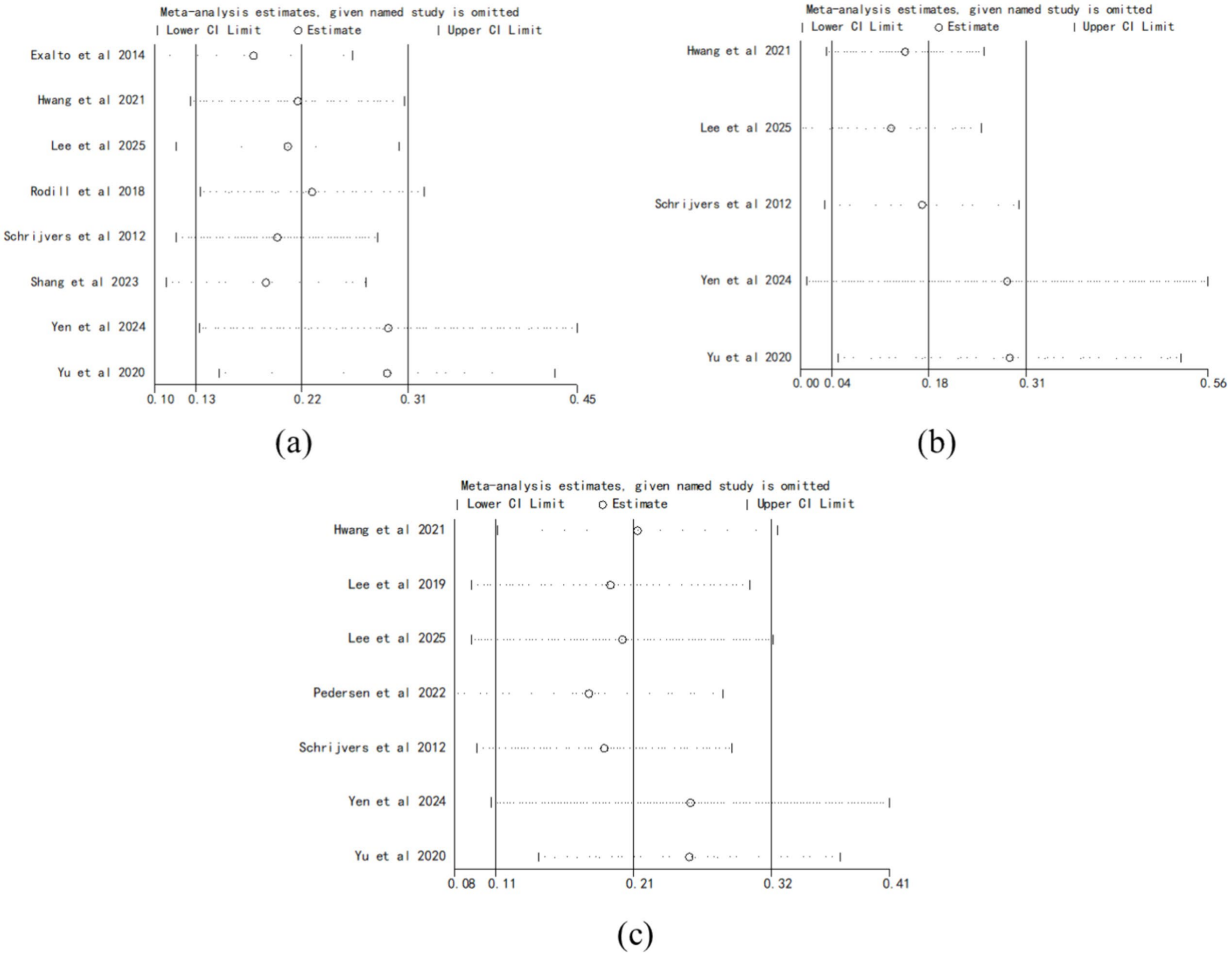
Sensitivity analysis. **(a)** All-cause dementia, **(b)** vascular dementia, and **(c)** Alzheimer’s disease.

The sensitivity analysis revealed that the pooled results of the meta-analysis regarding risk associations between diabetic retinopathy patients and ACD, AD, and VD remained unchanged after sequentially excluding individual studies, demonstrating high stability of the overall meta-analysis findings (Figure 5).

### Publication bias

**Figure 6:**
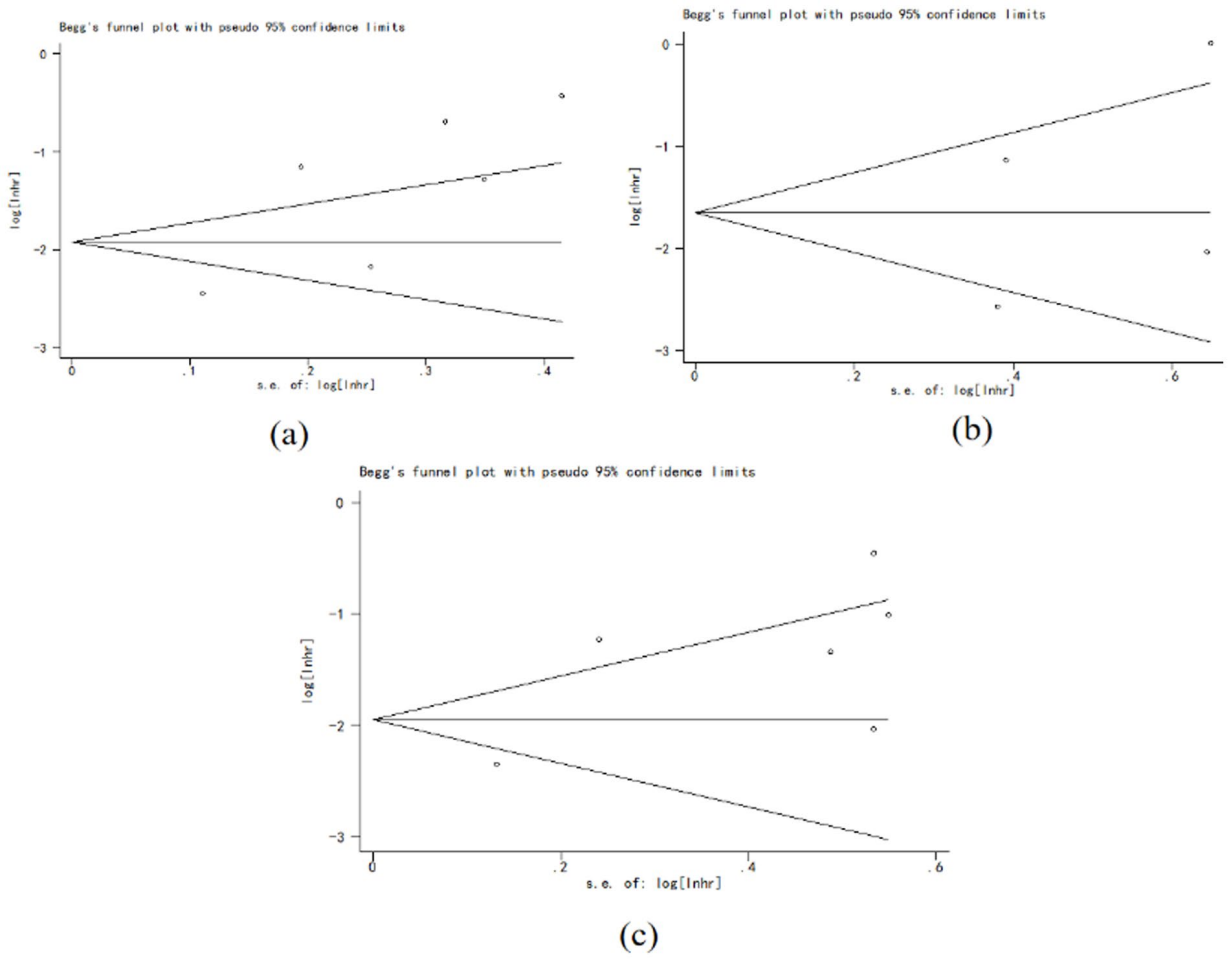
Begg’s funnel plot. **(a)** All-cause dementia, **(b)** vascular dementia, and **(c)** Alzheimer’s disease.

Begg’s and Egger’s regression tests were performed to evaluate potential publication bias. The results revealed no significant publication bias for any of the pooled analyses (Table 2; Figure 6).

## Discussion

This systematic review and meta-analysis comprehensively evaluated the associations between DR and the risks of ACD, AD, and VD. The pooled findings demonstrated that DR significantly increases the risk of ACD, AD, and VD. These results provide important insights into the potential link between diabetic microvascular complications and cognitive dysfunction. As a critical microvascular complication of diabetes, DR may serve as a measurable indicator of dementia susceptibility, suggesting a potential mechanistic role for systemic microvascular impairment in cognitive decline. Collectively, these findings highlight the need to optimize holistic management strategies for patients with diabetes. Beyond routine glycemic control and complication management, intensified cognitive function monitoring and regular assessment are warranted to facilitate early identification and timely intervention of dementia risk in diabetic populations.

Multiple cohort studies have investigated the association between DR and the risk of dementia (24, 30, 31). Several previous studies (22–24) have reported that DR patients exhibit a significantly elevated risk of dementia, which is consistent with the findings of the present meta-analysis. A prior meta-analysis by Wu et al. (32) reported a pooled HR of approximately 1.34 for the association between DR and cognitive impairment, which is highly comparable with our results and further strengthens the evidence that DR confers an increased risk of cognitive dysfunction. Furthermore, our subgroup analysis stratified by APOE ε4 adjustment status demonstrated that DR remained a significant risk factor for ACD, AD, and VD, regardless of APOE ε4 adjustment, and the findings were consistent across other stratified subgroups. Moreover, sensitivity analysis confirmed the robustness and stability of the overall pooled results, further validating the reliability of our meta-analytic estimates.

As a hallmark diabetic microvascular complication, DR is biologically linked to dementia via shared pathological mechanisms, including chronic inflammation, oxidative stress, impaired cerebral vascular autoregulation, and dysfunction of the blood–retinal and blood–brain barriers (33–36). The retina and the brain share highly similar embryonic origins, anatomical structures, and vascular properties, rendering retinal microvascular abnormalities a credible biomarker for reflecting cerebral microvascular health (37). Notably, our study observed a consistent risk-enhancing effect of DR on ACD, AD, and VD, with all pooled HRs ranging narrowly from 1.20 to 1.24.

AD and VD are etiologically heterogeneous disorders: AD is predominantly characterized by amyloid deposition and neurofibrillary tangle formation, whereas VD arises primarily from cerebrovascular lesions. Accumulating epidemiological evidence has further indicated that diabetes exerts a stronger and more consistent effect on vascular diseases (typical HR: 1.5–2.5 or higher) than on AD (typical HR: ≤1.5) (17–19). Accordingly, the comparable DR-associated risk elevations across AD and VD observed in our study contrast intriguingly with the inherent etiological heterogeneity of the two dementia subtypes and the divergent impacts of diabetes on different dementia types. This phenomenon warrants an in-depth mechanistic exploration.

The consistent risk-increasing effect of DR on both AD and VD observed in the present study is most likely attributable to DR serving as a robust indicator of systemic microvascular impairment. Such microvascular damage may accelerate the pathological progression of dementia through shared vascular dysfunction pathways, regardless of the distinct etiologies of AD and VD. Importantly, this finding does not negate the inherent etiological heterogeneity between the two dementia subtypes. Instead, it underscores the pivotal role of microvascular integrity in preserving cognitive function and suggests that severe diabetic microvascular complications—particularly DR—may serve as a critical clinical biomarker for generalized cognitive decline across all dementia subtypes among individuals with diabetes.

Accumulating evidence from previous studies (22, 36) has demonstrated that the association between DR and dementia remains significant even after adjusting for conventional cardiovascular risk factors and diabetes duration or severity. This suggests that DR may contribute to dementia through pathological mechanisms independent of systemic vascular dysfunction, including blood–retinal barrier disruption, chronic inflammation, and insulin resistance. Nevertheless, several limitations regarding residual confounding should be acknowledged. Current evidence fails to fully account for unmeasured or incompletely adjusted covariates, such as glycemic control status and the burden of other microvascular complications, which may lead to overestimation or underestimation of the pooled HRs (approximately 1.20–1.24) in the present analysis. Longer diabetes duration is closely correlated with both DR prevalence and elevated dementia risk; inadequate adjustment for this variable may overestimate the independent effect of DR. Conversely, unmeasured socioeconomic status, as a potential protective factor for cognitive function, may result in underestimation of effect sizes.

Although standardized covariate adjustment across all included studies was not feasible in this meta-analysis, we performed subgroup analyses stratified by APOE ε4 adjustment status to evaluate the influence of varying adjustment strategies on the pooled outcomes. The positive association between DR and dementia remained robust regardless of APOE ε4 adjustment (Table 3), indicating that the primary findings were not driven by any single confounding factor. Furthermore, the sensitivity analysis did not identify any directional changes in the overall results, confirming that no individual study with a distinct covariate adjustment set dominated the pooled effect estimate.

Future studies are warranted to adopt more rigorous analytical designs, such as Mendelian randomization and propensity score matching, and to collect comprehensive confounding variables—including glycemic control, diabetes duration, multiple microvascular complications, and inflammatory biomarkers. Such well-designed studies will help elucidate the independent causal relationship between DR and dementia risk.

DR is accompanied by retinal insulin resistance, defined as reduced insulin sensitivity in retinal cells, which subsequently impairs retinal metabolism and physiological function (38, 39). This metabolic abnormality further disturbs cerebral energy metabolism and neurotransmitter signaling, thereby inducing neuronal damage and apoptosis in the brain (40). Insulin resistance also upregulates vascular endothelial growth factor (VEGF) expression. Although elevated VEGF promotes retinal neovascularization, these newly formed vessels are structurally and functionally defective, with high susceptibility to rupture and hemorrhage, consequently exacerbating retinal pathological injury (41, 42). The brain is primarily dependent on glucose for energy supply; thus, insulin resistance reduces glucose uptake and utilization in cerebral neurons, leading to energy insufficiency and impaired neuronal function. Moreover, insulin resistance disrupts neurotransmitter synthesis and secretion, particularly diminishing acetylcholine production (43, 44). Given that acetylcholine is essential for learning, memory, and cognitive regulation, its deficiency substantially contributes to cognitive impairment (43).

In addition, chronic inflammation during DR progression damages retinal vascular endothelial cells and disrupts the integrity of the blood–retinal barrier (45, 46). Persistent inflammatory stimulation activates microglia and triggers the release of pro-inflammatory mediators, which further aggravate neuronal damage (47). Excessive reactive oxygen species produced by DR-related oxidative stress cause injury to retinal blood vessels and neurons, while also inducing cerebral neuronal damage, amyloid-β deposition, and neurofibrillary tangle formation in the brain (48, 49). Furthermore, DR-related microvascular alterations, including retinal microvascular narrowing, occlusion, and pathological neovascularization, impair cerebral blood perfusion and nutrient delivery, resulting in cerebral ischemia, hypoxia, and subsequent neuronal injury and loss (50, 51). Collectively, these synergistic metabolic, inflammatory, oxidative, and microvascular aberrations comprehensively elevate the risk of dementia in DR patients.

Several inherent limitations of the present meta-analysis should be acknowledged when interpreting its findings. All included studies were observational cohort studies. Despite rigorous quality evaluation and sensitivity analyses, observational designs cannot fully eliminate residual confounding, thereby limiting the ability to establish definitive causal relationships. Mendelian randomization (MR), a robust genetic causal inference approach, has recently advanced understanding of the associations between diabetic complications and dementia. By leveraging genetic variants as instrumental variables to mimic randomized controlled trial designs, MR effectively minimizes confounding and reverses causality biases inherent in conventional observational research (52). Previous MR studies had proposed a potential causal link between type 2 diabetes and AD risk, although the relevant evidence remains inconsistent (52–54). Notably, dedicated MR evidence focusing specifically on DR and distinct dementia subtypes remains limited. Finally, the generalizability of our findings should be interpreted with caution. Most included studies were derived from specific health systems or national registries in the United States, South Korea, Denmark, Netherlands, and China. Although these studies encompass both Asian and non-Asian populations, the results may not be directly applicable to other ethnic groups (e.g., African, Latin American, or Middle Eastern populations) or to community-based settings with different diabetes management practices, healthcare access, or socioeconomic conditions. Future studies on diverse ethnic and community populations are needed to validate the external validity of our findings.

Compared with individual MR analyses, meta-analyses can synthesize available cohort evidence and enhance statistical power to detect cross-sectional associations. Nevertheless, such analyses primarily demonstrate correlation rather than causality. Accordingly, the present findings should be interpreted as robust epidemiological evidence of a clinical association, which provides valuable hypotheses for future rigorous causal investigations, including high-quality MR studies and large-scale prospective cohorts. Future research integrating multi-omics data and genetic tools is warranted to clarify whether the observed association between DR and dementia reflects a direct causal effect, a shared pathophysiological foundation, or merely a marker of aggravated disease severity.

## Conclusion

In conclusion, this meta-analysis demonstrates that DR is significantly associated with elevated risks of ACD, AD, and VD. Clinically, intensified early screening and long-term cognitive monitoring are strongly recommended for DR patients and should be incorporated into routine diabetes management. Given the inherent limitations in the quantity and quality of the currently available evidence, further validation based on large-scale, high-quality prospective cohort studies is warranted to consolidate and refine the present conclusions.

## Data Availability

The datasets presented in this study can be found in online repositories. The names of the repository/repositories and accession number(s) can be found in the article/Supplementary material.

## References

[1] Zhang B Zhou L Chen K Fang X Li Q Gao Z . Investigation on phenomics of traditional Chinese medicine from the diabetes. Phenomics. (2024) 4:257–68. doi: 10.1007/s43657-023-00146-6, 39398423 PMC11467137

[2] (NCD-RisC) NRFC. Worldwide trends in diabetes prevalence and treatment from 1990 to 2022: a pooled analysis of 1108 population-representative studies with 141 million participants. Lancet. (2024) 404:2077–93. doi: 10.1016/s0140-6736(24)02317-1, 39549716 PMC7616842

[3] Zhou TY Tian N Li L Yu R. Iridoids modulate inflammation in diabetic kidney disease: a review. J Integr Med. (2024) 22:210–22. doi: 10.1016/j.joim.2024.03.010, 38631983

[4] Zakir M Ahuja N Surksha MA Sachdev R Kalariya Y Nasir M . Cardiovascular complications of diabetes: from microvascular to macrovascular pathways. Cureus. (2023) 15:e45835. doi: 10.7759/cureus.45835, 37881393 PMC10594042

[5] Chen KY Chan HC Chan CM. Association between omega-3 fatty acid intake and risk of diabetic retinopathy: a systematic review and meta-analysis. J Nutr Health Aging. (2025) 29:100632. doi: 10.1016/j.jnha.2025.100632, 40987202 PMC12492007

[6] Qian HY Wei XH Huang JO. Inflammatory mechanisms in diabetic retinopathy: pathogenic roles and therapeutic perspectives. Am J Transl Res. (2025) 17:6262–74. doi: 10.62347/gbfo5856, 40950276 PMC12432744

[7] Teo ZL Tham YC Yu M Chee ML Rim TH Cheung N . Global prevalence of diabetic retinopathy and projection of burden through 2045: systematic review and meta-analysis. Ophthalmology. (2021) 128:1580–91. doi: 10.1016/j.ophtha.2021.04.027, 33940045

[8] Lin HT Zheng CM Tsai CH Chen CL Chou YC Zheng JQ . The association between diabetic retinopathy and macular degeneration: a nationwide population-based study. Biomedicine. (2024) 12:727. doi: 10.3390/biomedicines12040727, 38672083 PMC11047965

[9] Mabala DS Stokholm L Andersen N Andresen J Bek T Heegaard S . Diabetic retinopathy as an independent marker of cardiovascular disease in type 1 diabetes: results from a nationwide longitudinal matched case-cohort study. Acta Ophthalmol. (2024) 102:635–42. doi: 10.1111/aos.16653, 38345204

[10] Thinggaard BS Stokholm L Davidsen JR Larsen MC Möller S Thykjær AS . Diabetic retinopathy is a predictor of chronic respiratory failure: a nationwide register-based cohort study. Heliyon. (2023) 9:e17342. doi: 10.1016/j.heliyon.2023.e17342, 37426795 PMC10329134

[11] Gao Y van Duijn C Littlejohns TJ Amin N. Neuroticism, omega-3 fatty acids, and risk of incident dementia. J Affect Disord. (2025) 388:119733. doi: 10.1016/j.jad.2025.119733, 40543620

[12] Kim J Han K Jung JH Oh SY Park KA Min JH. Optic neuritis as a link between autoimmunity and dementia risk. Commun Med. (2025) 5:335. doi: 10.1038/s43856-025-01050-y, 40770492 PMC12328802

[13] Huang T Beydoun MA Kianersi S Redline S Launer LJ. Multi-dimensional sleep health and dementia risk: a prospective study in the UK Biobank. BMC Med. (2025) 23:410. doi: 10.1186/s12916-025-04251-3, 40624639 PMC12235804

[14] Seo DH Kim M Cho Y Ahn SH Hong S Kim SH. Association between age at diagnosis of type 2 diabetes and subsequent risk of dementia and its major subtypes. J Clin Med. (2024) 13:1–13. doi: 10.3390/jcm13154386, 39124653 PMC11313191

[15] Thomassen JQ Tolstrup JS Benn M Frikke-Schmidt R. Type-2 diabetes and risk of dementia: observational and Mendelian randomisation studies in 1 million individuals. Epidemiol Psychiatr Sci. (2020) 29:e118. doi: 10.1017/s2045796020000347, 32326995 PMC7214711

[16] Wang M Fan C Han Y Wang Y Cai H Zhong W . Associations of modifiable dementia risk factors with dementia and cognitive decline: evidence from three prospective cohorts. Front Public Health. (2025) 13:1–14. doi: 10.3389/fpubh.2025.1529969, 39882349 PMC11774717

[17] Dybjer E Kumar A Nägga K Engström G Mattsson-Carlgren N Nilsson PM . Polygenic risk of type 2 diabetes is associated with incident vascular dementia: a prospective cohort study. Brain Commun. (2023) 5:1–13. doi: 10.1093/braincomms/fcad054, 37091584 PMC10118265

[18] Huang CC Chung CM Leu HB Lin LY Chiu CC Hsu CY . Diabetes mellitus and the risk of Alzheimer's disease: a nationwide population-based study. PLoS One. (2014) 9:e87095. doi: 10.1371/journal.pone.0087095, 24489845 PMC3906115

[19] Kadohara K Sato I Kawakami K. Diabetes mellitus and risk of early-onset Alzheimer's disease: a population-based case-control study. Eur J Neurol. (2017) 24:944–9. doi: 10.1111/ene.13312, 28503814

[20] Pedersen FN Stokholm L Pouwer F Hass Rubin K Peto T Frydkjær-Olsen U . Diabetic retinopathy predicts risk of Alzheimer's disease: a Danish registry-based Nationwide cohort study. J Alzheimers Dis. (2022) 86:451–60. doi: 10.3233/jad-215313, 35068460 PMC9028615

[21] Rodill LG Exalto LG Gilsanz P Biessels GJ Quesenberry CP Jr Whitmer RA. Diabetic retinopathy and dementia in type 1 diabetes. Alzheimer Dis Assoc Disord. (2018) 32:125–30. doi: 10.1097/wad.0000000000000230, 29261519 PMC5963957

[22] Exalto LG Biessels GJ Karter AJ Huang ES Quesenberry CP Jr Whitmer RA. Severe diabetic retinal disease and dementia risk in type 2 diabetes. J Alzheimers Dis. (2014) 42:S109–17. doi: 10.3233/jad-132570, 24625797 PMC4373321

[23] Lee SH Hwang G Lee DY Jeon JY Kwag SJ Sohn SY . Prediction of diabetic retinopathy using machine learning and its association with dementia risk in older adults with type 2 diabetes mellitus. Diabetes Res Clin Pract. (2025) 226:112378. doi: 10.1016/j.diabres.2025.112378, 40675255

[24] Shang X Zhu Z Huang Y Zhang X Wang W Shi D . Associations of ophthalmic and systemic conditions with incident dementia in the UK biobank. Br J Ophthalmol. (2023) 107:275–82. doi: 10.1136/bjophthalmol-2021-319508, 34518160

[25] Hwang PH Longstreth WT Jr Thielke SM Francis CE Carone M Kuller LH . Ophthalmic conditions associated with dementia risk: the cardiovascular health study. Alzheimers Dement. (2021) 17:1442–51. doi: 10.1002/alz.12313, 33788406 PMC8527838

[26] Zhu Z Yuan X Zheng Y Dou B Liu L Loh PY . Effectiveness of acupuncture in managing aromatase inhibitor-related arthralgia in breast cancer: a systematic review and meta-analysis. Acupunct Herb Med. (2025) 5:352–65. doi: 10.1097/HM9.0000000000000172, 42290303

[27] Zhang A Li J He T Xie H Mou X Yeung TC . Efficacy and safety of acupuncture in treating low back and pelvic girdle pain during pregnancy: a systematic review and meta-analysis of randomized controlled trials. Acupunct Herbal Med. (2024) 4:346–57. doi: 10.1097/hm9.0000000000000093

[28] Lee CS Larson EB Gibbons LE Lee AY McCurry SM Bowen JD . Associations between recent and established ophthalmic conditions and risk of Alzheimer's disease. Alzheimers Dement. (2019) 15:34–41. doi: 10.1016/j.jalz.2018.06.2856, 30098888 PMC6333518

[29] Schrijvers EM Buitendijk GH Ikram MK Koudstaal PJ Hofman A Vingerling JR . Retinopathy and risk of dementia: the Rotterdam study. Neurology. (2012) 79:365–70. doi: 10.1212/WNL.0b013e318260cd7e, 22786586 PMC3400091

[30] Yen YH Yen FS Ko FS Wei JC Huang Y Yu TS . Microvascular disease and its association with dementia in patients with type 2 diabetes: a nationwide cohort study in Taiwan. Diabetes Obes Metab. (2024) 26:5399–407. doi: 10.1111/dom.15908, 39210562

[31] Yu JH Han K Park S Cho H Lee DY Kim JW . Incidence and risk factors for dementia in type 2 diabetes mellitus: a nationwide population-based study in Korea. Diabetes Metab J. (2020) 44:113–24. doi: 10.4093/dmj.2018.0216, 31769236 PMC7043975

[32] Wu M Mei F Hu K Feng L Wang Z Gao Q . Diabetic retinopathy and cognitive dysfunction: a systematic review and meta-analysis. Acta Diabetol. (2022) 59:443–59. doi: 10.1007/s00592-021-01829-0, 35112186

[33] Rahmati M Smith L Lee H Boyer L Fond G Yon DK . Associations between vision impairment and eye diseases with dementia, dementia subtypes and cognitive impairment: an umbrella review. Ageing Res Rev. (2024) 101:102523. doi: 10.1016/j.arr.2024.102523, 39369799

[34] Liu YJ Zhao JY Han WW Yang HH Wu XB Xie F . Microvascular burden and long-term risk of stroke and dementia in type 2 diabetes mellitus. J Affect Disord. (2024) 354:68–74. doi: 10.1016/j.jad.2024.03.053 38479499

[35] Little K Llorián-Salvador M Scullion S Hernández C Simó-Servat O Del Marco A . Common pathways in dementia and diabetic retinopathy: understanding the mechanisms of diabetes-related cognitive decline. Trends Endocrinol Metab. (2022) 33:50–71. doi: 10.1016/j.tem.2021.10.008 34794851

[36] Lee CS Krakauer C Su YR Walker RL Blazes M McCurry SM . Diabetic retinopathy and dementia association, beyond diabetes severity. Am J Ophthalmol. (2023) 249:90–8. doi: 10.1016/j.ajo.2022.12.003, 36513155 PMC10106379

[37] Ehtewish H Arredouani A El-Agnaf O. Diagnostic, prognostic, and mechanistic biomarkers of diabetes mellitus-associated cognitive decline. Int J Mol Sci. (2022) 23:6144. doi: 10.3390/ijms23116144, 35682821 PMC9181591

[38] Järgen P Dietrich A Herling AW Hammes HP Wohlfart P. The role of insulin resistance in experimental diabetic retinopathy-genetic and molecular aspects. PLoS One. (2017) 12:e0178658. doi: 10.1371/journal.pone.0178658, 28575111 PMC5456117

[39] Xu YX Pu SD Zhang YT Tong XW Sun XT Shan YY . Insulin resistance is associated with the presence and severity of retinopathy in patients with type 2 diabetes. Clin Experiment Ophthalmol. (2024) 52:63–77. doi: 10.1111/ceo.14344, 38130181

[40] Zagare A Kurlovics J Almeida C Ferrante D Frangenberg D Vitali A . Insulin resistance compromises midbrain organoid neuronal activity and metabolic efficiency predisposing to Parkinson's disease pathology. J Tissue Eng. (2025) 16:20417314241295928. doi: 10.1177/20417314241295928, 39882547 PMC11775974

[41] Arrigo A Aragona E Bandello F. Vegf-targeting drugs for the treatment of retinal neovascularization in diabetic retinopathy. Ann Med. (2022) 54:1089–111. doi: 10.1080/07853890.2022.2064541, 35451900 PMC9891228

[42] Callan A Heckman J Tah G Lopez S Valdez L Tsin A. VEGF in diabetic retinopathy and age-related macular degeneration. Int J Mol Sci. (2025) 26:4992. doi: 10.3390/ijms26114992, 40507802 PMC12154301

[43] de la Monte SM. Type 3 diabetes is sporadic Alzheimer′s disease: mini-review. Eur Neuropsychopharmacol. (2014) 24:1954–60. doi: 10.1016/j.euroneuro.2014.06.008, 25088942 PMC4444430

[44] He Y Sun M Qu M Lu Y Yang H Wang R . Brain insulin signaling pathway regulation of hippocampal neuroplasticity in neurocognitive disorders: mechanisms and therapeutic implications. J Integr Neurosci. (2025) 24:39446. doi: 10.31083/jin39446, 40919624

[45] Tang L Xu GT Zhang JF. Inflammation in diabetic retinopathy: possible roles in pathogenesis and potential implications for therapy. Neural Regen Res. (2023) 18:976–82. doi: 10.4103/1673-5374.355743, 36254977 PMC9827774

[46] Monickaraj F Acosta G Cabrera AP Das A. Transcriptomic profiling reveals chemokine CXCL1 as a mediator for neutrophil recruitment associated with blood-retinal barrier alteration in diabetic retinopathy. Diabetes. (2023) 72:781–94. doi: 10.2337/db22-0619, 36930735 PMC10202768

[47] Whitney NP Eidem TM Peng H Huang Y Zheng JC. Inflammation mediates varying effects in neurogenesis: relevance to the pathogenesis of brain injury and neurodegenerative disorders. J Neurochem. (2009) 108:1343–59. doi: 10.1111/j.1471-4159.2009.05886.x, 19154336 PMC2707502

[48] Mrowicka M Mrowicki J Majsterek I. Relationship between biochemical pathways and non-coding RNAs involved in the progression of diabetic retinopathy. J Clin Med. (2024) 13:292. doi: 10.3390/jcm13010292, 38202299 PMC10779474

[49] Weinstock M. Role of oxidative stress and neuroinflammation in the etiology of Alzheimer's disease: therapeutic options. Antioxidants. (2025) 14:769. doi: 10.3390/antiox14070769, 40722873 PMC12291978

[50] Upadhyay T Prasad R Mathurkar S. A narrative review of the advances in screening methods for diabetic retinopathy: enhancing early detection and vision preservation. Cureus. (2024) 16:e53586. doi: 10.7759/cureus.53586, 38455792 PMC10918290

[51] Sweeney MD Zhao Z Montagne A Nelson AR Zlokovic BV. Blood-brain barrier: from physiology to disease and back. Physiol Rev. (2019) 99:21–78. doi: 10.1152/physrev.00050.2017, 30280653 PMC6335099

[52] Liu D Cao H Baranova A Xu C Zhang F. Opposite causal effects of type 2 diabetes and metformin on Alzheimer's disease. J Prev Alzheimers Dis. (2025) 12:100129. doi: 10.1016/j.tjpad.2025.100129, 40064559 PMC12434270

[53] Li C Qian H Feng L Li M. Causal association between type 2 diabetes mellitus and Alzheimer's disease: a two-sample Mendelian randomization study. J Alzheimer's Dis Rep. (2024) 8:945–57. doi: 10.3233/adr-240053, 39114544 PMC11305840

[54] Han S Lelieveldt T Sturkenboom M Biessels GJ Ahmadizar F. Evaluating the causal association between type 2 diabetes and Alzheimer's disease: a two-sample Mendelian randomization study. Biomedicine. (2025) 13:1095. doi: 10.3390/biomedicines13051095, 40426922 PMC12108868

