# Supplementary material for: Diabetic retinopathy and the risk of all-cause dementia, Alzheimer’s disease, and vascular dementia: a systematic review and meta-analysis
Source: Front Med (Lausanne). 2026 Jun 30;13:1716648. doi: 10.3389/fmed.2026.1716648 (PMC13364685; doi:10.3389/fmed.2026.1716648)
Supplement: Supplementary file 1 [file Data_Sheet_1.docx]

| **Supplementary Table S1. Search strategy** | |
| --- | --- |
| Pubmed | ("Diabetic Retinopathy"[MeSH] OR "Diabetic Retinopathies"[Title/Abstract] OR "Retinopathies, Diabetic"[Title/Abstract] OR "Retinopathy, Diabetic"[Title/Abstract]) AND ("Dementia"[MeSH] OR "Dementias"[Title/Abstract] OR "Amentia"[Title/Abstract] OR "Amentias"[Title/Abstract] OR "Senile Paranoid Dementia"[Title/Abstract] OR "Familial Dementia"[Title/Abstract] OR "Alzheimer Disease"[MeSH] OR "Alzheimer Syndrome"[Title/Abstract] OR "Alzheimer-Type Dementia"[Title/Abstract] OR "Alzheimer Type Dementia"[Title/Abstract] OR "Dementia, Alzheimer-Type"[Title/Abstract] OR "Alzheimer's Diseases"[Title/Abstract] OR "Alzheimer Dementia"[Title/Abstract] OR "Alzheimer's Disease"[Title/Abstract] OR "Dementia, Senile"[Title/Abstract] OR "Senile Dementia"[Title/Abstract] OR "Dementia, Alzheimer Type"[Title/Abstract] OR "Alzheimer Type Senile Dementia"[Title/Abstract] OR "Primary Senile Degenerative Dementia"[Title/Abstract] OR "Presenile Dementia"[Title/Abstract] OR "Early Onset Alzheimer Disease"[Title/Abstract] OR "Late Onset Alzheimer Disease"[Title/Abstract] OR "Familial Alzheimer Disease"[Title/Abstract] OR "Dementia, Vascular"[MeSH] OR "Vascular Dementias"[Title/Abstract] OR "Vascular Dementia"[Title/Abstract] OR "Arteriosclerotic Dementia"[Title/Abstract] OR "Binswanger Disease"[Title/Abstract] OR "Subcortical Vascular Dementia"[Title/Abstract] OR "Acute Onset Vascular Dementia"[Title/Abstract]) AND  ("Cohort Studies"[MeSH] OR "Cohort Study"[Title/Abstract] OR "Studies, Cohort"[Title/Abstract] OR "Study, Cohort"[Title/Abstract] OR "Concurrent Studies"[Title/Abstract] OR "Closed Cohort Studies"[Title/Abstract] OR "Historical Cohort Studies"[Title/Abstract] OR "Incidence Studies"[Title/Abstract] OR "Cohort Analysis"[Title/Abstract] OR "Birth Cohort Studies"[Title/Abstract] OR "Observational Study"[Publication Type] OR "Observational Studies as Topic"[MeSH] OR "Natural Experiments as Topic"[MeSH] OR "Naturalistic Observation Studies as Topic"[MeSH] OR "Longitudinal Studies"[MeSH] OR "Follow-Up Studies"[MeSH] OR "Prospective Studies"[MeSH] OR "Retrospective Studies"[MeSH] OR "Longitudinal Study"[Title/Abstract] OR "Follow-Up Study"[Title/Abstract] OR "Prospective Study"[Title/Abstract] OR "Retrospective Study"[Title/Abstract]) |
| Embase | ('diabetic retinopathy'/exp OR 'diabetic retinopathy' OR 'diabetic retinopathies') AND ('dementia'/exp OR 'dementia' OR 'alzheimer disease'/exp OR 'alzheimer disease' OR 'vascular dementia'/exp OR 'vascular dementia') AND ('cohort analysis'/exp OR 'longitudinal study'/exp OR 'prospective study'/exp OR 'retrospective study'/exp OR 'cohort stud*' OR 'longitudinal stud*') |
| Web of Science | TS=("diabetic retinopathy" OR "diabetic retinopathies" OR "retinopathy diabetic" OR "retinopathies diabetic")AND TS=("dementia" OR "dementias" OR "amentia" OR "senile paranoid dementia" OR "familial dementia" OR "Alzheimer disease" OR "Alzheimer syndrome" OR "Alzheimer-type dementia" OR "Alzheimer type dementia" OR "Alzheimer's disease" OR "Alzheimer dementia" OR "senile dementia" OR "presenile dementia" OR "early onset Alzheimer" OR "late onset Alzheimer" OR "familial Alzheimer" OR "vascular dementia" OR "vascular dementias" OR "arteriosclerotic dementia" OR "Binswanger disease" OR "subcortical vascular dementia" OR "acute onset vascular dementia") AND TS=("cohort study" OR "cohort studies" OR "longitudinal study" OR "longitudinal studies" OR "prospective study" OR "prospective studies" OR "retrospective study" OR "retrospective studies" OR "observational study" OR "observational studies" OR "follow-up study" OR "follow-up studies" OR "incidence study" OR "incidence studies" OR "cohort analysis") |

| **Supplementary Materials Table S2.The literature included in the full-text screening process** | | | |
| --- | --- | --- | --- |
| ID | Literature citation information | Whether to include in the meta-analysis | Reasons for excluding the literature |
| 1 | Diabetic Retinopathy Predicts Risk of Alzheimer's Disease: A Danish Registry-Based Nationwide Cohort Study. | Yes |  |
| 2 | Diabetic Retinopathy and Dementia in Type 1 Diabetes. | Yes |  |
| 3 | Severe Diabetic Retinal Disease and Dementia Risk in Type 2 Diabetes | Yes |  |
| 4 | Prediction of Diabetic Retinopathy Using Machine Learning and Its Association with Dementia Risk in Older Adults with Type 2 Diabetes Mellitus | Yes |  |
| 5 | Associations of Ophthalmic and Systemic Conditions with Incident Dementia in the Uk Biobank | Yes |  |
| 6 | Ophthalmic Conditions Associated with Dementia Risk: The Cardiovascular Health Study | Yes |  |
| 7 | Associations between Recent and Established Ophthalmic Conditions and Risk of Alzheimer's Disease | Yes |  |
| 8 | Retinopathy and Risk of Dementia: The Rotterdam Study | Yes |  |
| 9 | Microvascular Disease and Its Association with Dementia in Patients with Type 2 Diabetes: A Nationwide Cohort Study in Taiwan | Yes |  |
| 10 | Incidence and Risk Factors for Dementia in Type 2 Diabetes Mellitus: A Nationwide Population-Based Study in Korea | Yes |  |
| 11 | Prediction of diabetic retinopathy using machine learning and its association with dementia risk in older adults with type 2 diabetes mellitus | No | Data cannot be extracted |
| 12 | Retinal signs and risk of incident dementia in the Atherosclerosis Risk in Communities study | No | Data cannot be extracted |
| 13 | Microvascular disease and its association with dementia in patients with type 2 diabetes: A nationwide cohort study in Taiwan | No | No outcome of interest |
| 14 | Microvascular burden and long-term risk of stroke and dementia in type 2 diabetes mellitus | No | No outcome of interest |
